# Socioeconomic and Environmental Risk Factors among Rheumatic Heart Disease Patients in Uganda

**DOI:** 10.1371/journal.pone.0043917

**Published:** 2012-08-27

**Authors:** Emmy Okello, Barbara Kakande, Elias Sebatta, James Kayima, Monica Kuteesa, Boniface Mutatina, Wilson Nyakoojo, Peter Lwabi, Charles K. Mondo, Richard Odoi-Adome, Freers Juergen

**Affiliations:** 1 Division of Cardiology, Department of Medicine, Makerere University, Kampala, Uganda; 2 Uganda Heart Institute, Mulago Hospital, Kampala, Uganda; 3 School of Pharmacy, Makerere University, Kampala, Uganda; 4 Sydney School of Public Health, Sydney, Australia; 5 College of Health Sciences, Makerere University, Kampala, Uganda; McGill University, Canada

## Abstract

**Background:**

Although low socioeconomic status, and environmental factors are known risk factors for rheumatic heart disease in other societies, risk factors for rheumatic heart disease remain less well described in Uganda.

**Aims and Objective:**

The objective of this study was to investigate the role of socio-economic and environmental factors in the pathogenesis of rheumatic heart disease in Ugandan patients.

**Methods:**

This was a case control study in which rheumatic heart disease cases and normal controls aged 5–60 years were recruited and investigated for socioeconomic and environmental risk factors such as income status, employment status, distance from the nearest health centre, number of people per house and space area per person.

**Results:**

486 participants (243 cases and 243 controls) took part in the study. Average age was 32.37+/−14.6 years for cases and 35.75+/−12.6 years for controls. At univariate level, Cases tended to be more overcrowded than controls; 8.0+/−3.0 versus 6.0+/−3.0 persons per house. Controls were better spaced at 25.2 square feet versus 16.9 for cases. More controls than cases were employed; 45.3% versus 21.1%. Controls lived closer to health centers than the cases; 4.8+/−3.8 versus 3.3+/−12.9 kilometers. At multivariate level, the odds of rheumatic heart disease was 1.7 times higher for unemployment status (OR = 1.7, 95% CI = 1.05–8.19) and 1.3 times higher for overcrowding (OR = 1.35, 95% CI = 1.1–1.56). There was interaction between overcrowding and longer distance from the nearest health centre (OR = 1.20, 95% CI = 1.05–1.42).

**Conclusion:**

The major findings of this study were that there was a trend towards increased risk of rheumatic heart disease in association with overcrowding and unemployment. There was interaction between overcrowding and distance from the nearest health center, suggesting that the effect of overcrowding on the risk of acquiring rheumatic heart disease increases with every kilometer increase from the nearest health center.

## Introduction

Rheumatic heart disease (RHD) remains a forgotten yet preventable disease in developing countries, with devastating consequences for young adults. [1] RHD and Acute Rheumatic Fever (ARF) affect about 16 million people worldwide, with over 70% in Sub-Saharan Africa where the prevalence has been found to be between 6.5 to 30 per1000 people. [2], [3], [4] Although no national prevalence data exists on RHD in Uganda, we have recently found a prevalence of 14 per 1000 cases among randomly selected primary school children in Kampala district, the country’s capital. [5], [6] Anecdotal data from a hospital based study done at Mulago National Referral Hospital ranked RHD as the commonest cause of heart disease in the 15 to 49 age group, and second commonest cause of heart disease overall, after hypertension. [7]

The pathophysiology of RHD is thought to involve the triad of host genetic makeup, Group A streptococcal virulence and environmental factors. However, environmental factors are thought to affect disease epidemiology the most. Acute rheumatic fever prevalence has been seen to go down as living conditions improve. [8], [9] A low socioeconomic status mainly characterized by poverty, illiteracy and unemployment has been associated with RHD, although a direct link between these factors and the disease has not been confirmed. [10], [11] While ARF/RHD has been described as a disease of poverty, its persistence in most developing countries, and reemergence in developed countries where it had nearly disappeared raises concern on the role of environmental and other factors, other than poverty, [12], [13] and the consequential increase in group A Streptococci transmission. Some environmental factors such as overcrowding and poor ventilation have been directly related to susceptibility to ARF and ultimately RHD. [14], [15], [16], [17]

Currently, no study done in Uganda has documented the role or extent of socioeconomic and environmental factors in the pathogenesis of RHD. The objective of this study therefore, was to investigate the role of socioeconomic and environmental factors in the pathogenesis of RHD in Ugandan patients. Specific attention has been paid to environmental factors such as overcrowding and distance from the nearest health centre.

## Methods

### Ethical Approval

Ethical approval for the study was provided by the Institutional Review Board of the School of Medicine, Makerere University College of Heath Sciences and permission confirmed through the Uganda National Council for Science and Technology (UNCST).

The study conformed to the principles outlined in the declaration of Helsinki. Each study participant aged 18 years and above gave a written informed consent. Parents/next of kin gave a written informed consent on behalf of the minors/children participants. Study participants or their next of kin were asked to fill a questionnaire of socioeconomic and environmental factorsAll data were kept in confidence with each participant assigned a unique identifier code.

### Study Design

This was an unmatched case control study in which 243 cases and 243 controls were recruited using systematic random sampling in Mulago National Referral Hospital.

### Study Participants

#### Sample selection

A random sample of cases and controls from a variety of geographical and social origins was identified at Mulago National Referral Hospital. Controls recruited for the study were selected using systemic random sampling from among people referred for cardiac evaluation for suspected heart disease but found to have normal echocardiograms.

### Eligibility and Exclusion Criteria

Cases were subjects aged 5 to 60 years, diagnosed with RHD based on history of acute rheumatic fever, clinical examination finding of a murmur and standard echocardiographic criteria. Only cases living at the current location for the last five years were considered. Cases with possible and probable RHD were excluded from the study. Comparable eligibility criteria for controls included subjects aged 5 to 60 years with no previous history of RHD and had normal echocardiograms and living at the current location for the last five years were considered. Controls with raised antitreptolysin O titres on laboratory evaluation were excluded.

### Echocardiography

Echocardiography was done according to standard criteria [18] Briefly, Standard transthoracic echocardiography (GE, Vivid 8, Chicago, USA) was performed where 2D, M-mode, colour and doppler modalities were done with images taken from parasternal long axis, parasternal short axis, apical four chamber, apical five chamber and subcostal views. Mitral stenosis was diagnosed by two-dimensional echocardiographic and hemodynamic evidence, as well as characteristic mitral valve morphology, including thickened mitral leaflets, cusps, commissures, anterior mitral leaflet doming in the long-axis view, anterior motion of the posterior mitral leaflet on M-mode echocardiography. Mitral valve area was graded based on planimetry and pressure half time methods. Mitral stenosis was graded as mild for valve area between 1.6–2.0 cm2, moderate for valve area 1.1–1.5 cm2, and severe for valve area <1.0 cm2. Aortic stenosis was diagnosed based on the presence of commissural fusion of the aortic leaflets, possible increased echogenicity along the leaflet edges, and systolic doming of the aortic leaflets. Valve area was assessed using planimetry and classified as mild (vale area >1.5 cm2), moderate (valve area 1–1.5 cm2) and severe if valve area was <1 cm2. Mitral and aortic regurgitation were assessed using quantitative methods.

### Socioeconomic and Environmental Status

The following parameters were documented; age, gender, level of education, monthly earning (in Uganda Shillings and later converted to United States dollars), number of people per house, distance from the nearest health center (kilometers) and space area per person (square feet).

### Level of Income

For standardization purposes, we used the modified Prasad classification of social status which divides individuals into 4 social strata including lower class (below a dollar a day) middle class, middle-high and highest class. [19] The Prasad classification was adapted to local conditions by converting the Indian Rupees to Uganda Shillings and to United States dollars. For study participants below 18 years, we used their mothers’ income as an estimate.

### Number of People Per House and Space Area

The number of people per house, and space area were determined to define overcrowding.

A household with over 8 people was classified as overcrowded. Similarly, basing on the American Public Health Association recommendation that in a shared household, each individual should have at least 90 square feet of space [20], we surveyed the homes of at least 100 cases and 100 controls (50%) of each study group, and determined the average house area per study group (120 square feet for controls versus 80 square feet for cases). We then divided the average house area of the control group, which was higher, by the number of people per house in the controls and cases study groups to determine space area per person, which was compared between the two groups.

### Distance from the Nearest Health Centre

Distance from the nearest health centre was determined to assess access to health care. Previous studies in developing countries have showed that distance of 5 kilometers or less to a health unit predicted better access to health care and therefore uptake of prevention programs such as vaccination. [21], [22]

### Statistical Analysis

Data was double-entered and stored in EPI data version 3.0. (EpiData Association, Odense M, Denmark) Analysis was done using STATA 10.0 statistical package. (Stata Corporation, College Station, TX, USA).

### Study Variables

With rheumatic heart disease status as an outcome (dependent) variable, the following were analyzed as independent variables; age (years), income level (Uganda Shillings/United States dollars), education status, number of people per house, space area per person (square feet), distance from the nearest health unit (kilometers) and employment status (yes/no).

### Data Analysis

At univariate analysis, the Chi square test was used for categorical variables and Student *t* and was used for continuous variables, respectively for statistical significance.

The following covariates that were significant at the univariate level (at p-values ≤0.2) were entered into the multivariate analysis; Age (years), education level, employment status (yes/no), number of people per house, space area per person (square feet) and distance from home to the nearest health centre (kilometer). Odds ratios were computed with 95% CI.

Logistic regression was used at multivariate analysis where covariates listed above were considered and tested for confounding and interaction. The backward likelihood ratio method was used to get the space area (sq feet) as most significant variable in the multivariate model (main predictor). To test for interaction, product terms were formed between space area (main predictor) and other basic variables in the model. Using the chunk test, where a fit of a model with all interaction terms included is compared with a fit of a model with none of the interaction terms [23]. The negative two log likelihood (−2LL) of the full model with interaction terms were included and the reduced model containing only basic variables were compared. Interaction was considered present when the difference between −2LL was significant (at p≤0.05) with a Chi-square test. Confounding would be considered present if the difference between crude and adjusted odds ratios would greater than or equal to 10%.

## Results

### Background Characteristics of Study Participants

A total of 486 (243 cases and 243 controls) participants took part in the study. About three quarters of cases (76.8%) and controls (72.4%) were females. The average age for controls was 35.75+/−12.6 years, higher than that of cases at 32.37+/−14.6 years. Table 1.

**Table 1:** Univariate Analysis of Socioeconomic Risk Factors for RHD.

Factor	Case of RHD N (%)	Control N (%)	UnadjustedOdds ratio	P-value	95% CI
**Sex**					
Female	186 (76.8)	176 (72.4)	1		
Male	57 (23.1)	67(27.6)	1.26	0.26	0.82–1.948
**Education level**					
No education	17 (6.2)	16 (6.6)	1		
Primary	114(47.1)	47 (19.0)	0.38	0.015	0.17–0.83
Secondary	79 (32.6)	108 (44.6)	1.28	0.523	0.59–2.74
Vocational	7 (2.9)	11 (4.5)	1.47	0.52	0.45–4.79
University	26 (10.7)	61 (25.2)	2.19	0.066	0.95–5.09
**Currently employed**					
No	191 (78.9)	133 (54.7)	1		
Yes	52 (21.1)	110 (45.3)	3.09	P<0.05	2.04–4.72
**Income (Uganda Shillings/US Dollar)**					
Less than 50000 (<25 USD)	27 (11.3)	10 (4.1)	1		
50000–99000 (25–49.5USD)	136 (57.1)	98 (40.3)	1.95	0.09	0.9–4.2
100000–199000 (50–99.5USD)	64 (24.8)	48 (19.7)	2.19	0.06	0.96–4.98
200000+(100 USD)	16 (6.7)	87 (35.8)	14.7	P<0.05	5.96–36.1
	**Mean+/−SD**	**Mean+/−SD**	**Mean diff**		
**Age (years)**	32.37+/−14.6	35.75+/−12.6	3.38	0.006	0.94–5.82

The average house area for controls was 120 square feet while that of cases was 80 square feet.

### Univariate Analysis

The majority of both cases (78.9%) and controls (72%) were unemployed with most earning between 25–49.5 United States dollars (USD) per month (cases-57%, controls-40%) Table 1. About a half of the cases (47%) and controls (44.6%) had attained primary and secondary level of education respectively (Table 1). The average distance from the nearest health center for cases (4.8 km) was slightly longer that the average distance from the nearest health center for controls (3.3 km) Table 2. While the average space area occupied by cases (16.9 sq ft) was less than that for controls (25.2 sq ft), the average number of people living in the house for cases (8.0) was more than that for controls (6.0) Table 2.

**Table 2:** Univariate Analysis of Environmental Risk Factors for RHD.

Variable	Cases (n = 243)	Controls (n = 243)	OR	P value	95% CI
**Space area (Sq feet)**	16.9+/−7.5	25.2+/−15.2	8.3	P<0.05	6.1–10.4
**Distance from the nearest health unit (Km)**	4.8+/−3.8	3.3+/−12.9	1.48	P = 0.045	0.2–3.2
**Number of people living in the house**	8.0+/−3.0	6.0+/−3.0	1.98	P<0.05	1.4–2.5

### Multivariate Analysis

At multivariate level, there was a trend towards increased risk of RHD, in association with unemployment status (OR = 1.7, 95% CI = 1.05–8.19), and overcrowding (OR = 1.35, 95% CI = 1.1–1.56). Distance from the nearest health center appeared to be protective from acquiring RHD (OR = 0.7, 95% CI = 0.6–0.87), although there was interaction when overcrowding was compared with distance from the nearest health center (OR = 1.20, 95% CI = 1.05–1.42), indicating that the effect of overcrowding on the risk of developing RHD increases with every kilometer increase in the distance from the nearest health center. (Table 3).

**Table 3:** Multivariate Analysis of Socioeconomic and Environmental Risk Factors for RHD.

Factor	Adjusted Odds ratio	P-value	95% CI
**Education level**			
No education	1		
Primary	0.57	0.231	0.22–1.43
Secondary	1.91	0.171	0.75–4.88
Vocational	1.8	0.405	0.45–7.21
University	2.93	0.05	1.04–8.19
**Currently employed**			
No	1.71	0.04	1.05–8.19
Yes	1		
**Distance from the nearest health unit (Km)**	0.7	0.001	0.61–0.87
Age (years)	3.38	0.006	0.94–5.82
**Space area (sq feet)**	1.35	0.04	1.10–1.56
Space area * Distance from nearest Health Unit	1.2	0.001	1.05–1.42

## Discussion

In this study designed to investigate the role and extent of socioeconomic and environmental risk factors among RHD patients in Uganda, we found a trend towards increased risk of RHD in association with overcrowding and unemployment. The effect of overcrowding on the risk of acquiring RHD increases with every kilometer increase from the nearest health center.

### Overcrowding

A previous study in Soweto, South Africa found a higher prevalence of RHD among suburban dwellers, usually the lower socioeconomic class, who also tended to live in overcrowded residences, while a survey in Congo Kinshasa, found that children from overcrowded suburban families had a higher prevalence of RHD irrespective of income and social classes of their parents [4], [24] More recently, Jaine et al, in an ecological study in New Zealand assessed 1249 cases of ARF diagnosed between 1996 and 2005 found a positive and significant association between ARF and overcrowding, although this study was limited by its design [25], (it was not case controlled making it impossible to draw conclusions on their findings). Our study highlights the importance of overcrowding in the pathogenesis of RHD, especially in the developing world where rapid urbanization and poor housing predisposes suburban dwellers to overcrowding and consequently ARF and RHD. [26] In addition, the reemergence of RHD in the intermountain region of the United States [27], [28] and a high prevalence in Italian army recruits, both developed nations in the 1980s, had strong association with overcrowding, rather than poverty. Societies where living conditions have improved, have recorded substantial decline in the prevalence of RHD. For instance, the recent decline in RHD in China and other emerging economies is apparently linked to economic development which translated into improved living conditions. However, it is worth noting that the decline in RHD was also associated with a total change in house designs. [29] City authorities moved away from overcrowded communal accommodation to large, spacious accommodation.

### Overcrowding and Distance from the Nearest Health Center

To the best of our knowledge, this is the first study assessing overcrowding and its interaction with distance from the nearest health center as a risk factor for RHD. Findings from our study suggest that the effect of overcrowding on the risk of acquiring ARF/RHD increased with every kilometer increase from the nearest health center, although on its own, longer distance travelled from a health center appeared to be protective (OR = 0.7, 95% CI = 0.6–0.87). These observations suggest that irrespective of distance from the nearest health center, an individual’s risk of acquiring RHD starts to increase when their household becomes overcrowded. Previous studies in Kenya, Pakistan and Yemen showed that for populations at risk, shorter distance from the nearest health center correlated with better access to vaccination services, control of malaria and other infectious diseases. [30], [31], [32]. For overcrowded households, at high risk of streptococcal pharyngitis, distance to a health center would determine access to prophylactic medication such as penicillin. The delay in accessing care as a result of longer distances from the health facility further raises the issue of ability of RHD cases to comply and go for frequent benzathine penicillin injections, an important component of treatment given to patients with RHD.

### Socioeconomic and Environmental Risk Factors for RHD

Although RHD has generally been associated with poverty [26], [33], [34] a direct link between RHD and poverty has not been found. In our, study we found no significant difference in income between cases and controls at the multivariate level. This absence of a difference could be due to the fact that in Uganda, most respondents associate income to earning from formal employment. And yet majority of people are in the informal sector, making it difficult to ascertain their actual level of income. Furthermore, in developing countries, income is generally low across the board, and a threshold might not have been reached where difference in social classes leads to a difference in disease risk factors. This is consistent with findings by Steer et al, who in a systemic review of RHD prevalence in developing countries and the role of environmental factors, assessed 24 studies conducted between 1976 and 1999, and concluded that a threshold level where higher socio-economic status is associated with reduced prevalence of RHD has not been reached in developing countries. Therefore, differences in prevalence between socio-economic groups in one area cannot be detected. [11] Dobson et al in a well designed case controlled study of socioeconomic and environmental risk factors for RHD in 80 cases and 80 controls in Fiji, found a trend toward increased risk of RHD in association with poor-quality housing and lower socioeconomic status but only maternal unemployment reached statistical significance. This study was however limited by a small sample size. [35]

### Study Limitations

First, we were not able to visit all the households of the study participants to ascertain the state of overcrowding, because of time and resources limitation. Secondly, we determined overcrowding based on residence in an area for the last 5 years, it would have been beneficial to know housing status of an individual for the past 15 years as the disease pathogenesis is known to take five to fifteen years.

### Conclusions

This study demonstrates that in Uganda, there is increased risk for RHD in association with overcrowding and unemployment. The effect of overcrowding on the risk of RHD seems to increase with every kilometer increase from a health center.

### Recommendations

Findings in the present study have implications for public health planners in Uganda and other developing nations in that in addition to primary and secondary prophylaxis of streptococcal throat infection, there is need to redesign housing plans that ensure that each individual in a shared household has at least 90 square feet of space to avoid overcrowding. In addition, mobile RHD screening clinics should be set up at schools and communities where health centers do not exist or are far from the population in order to increase accessibility to screening and other prevention services.
