# Correction: Socioeconomic and Environmental Risk Factors among Rheumatic Heart Disease Patients in Uganda

**DOI:** 10.1371/annotation/c6d4b7b9-bf5f-4d99-8df7-027ce99daf50

**Published:** 2013-12-16

**Authors:** Emmy Okello, Barbara Kakande, Elias Sebatta, James Kayima, Monica Kuteesa, Boniface Mutatina, Wilson Nyakoojo, Peter Lwabi, Charles K. Mondo, Richard Odoi-Adome, Freers Juergen

A statistic- the P-value for age- in Tables 1 and 3 is incorrect. The correct P-value is: 0.06.

